# Health-related quality of life in psychiatric outpatients: a cross-sectional study of associations with symptoms, diagnoses, and employment status

**DOI:** 10.1007/s11136-024-03748-3

**Published:** 2024-08-07

**Authors:** Audun Havnen, Martin Schevik Lindberg, Jakob Lundqvist, Martin Brattmyr, Odin Hjemdal, Stian Solem

**Affiliations:** 1 Department of Psychology, Norwegian University of Science and Technology, Trondheim, NO-7491 Norway; 2 Division of Psychiatry, Nidaros Community Mental Health Centre, St. Olav’s University Hospital, Trondheim, Norway; 3 Health and welfare, Trondheim Municipality, Norway

**Keywords:** Health-related quality of life, EQ-5D-5L, Psychiatric outpatient treatment, Specialist health care

## Abstract

**Background:**

This cross-sectional study aimed to explore health-related quality of life (HRQoL) in a large heterogeneous patient sample seeking outpatient treatment at a specialist mental health clinic.

**Method:**

A sample of 1947 patients with common mental disorders, including depressive-, anxiety-, personality-, hyperkinetic- and trauma-related disorders, completed the EuroQoL 5-Dimension 5-Level (EQ-5D-5L) to assess HRQoL. We investigated clinical and sociodemographic factors associated with the EQ-5D index and the EQ Visual Analogue Scale (VAS) using regression analyses.

**Results:**

The sample reported lower HRQoL compared with the general population and primary mental health care patients. Sick leave, disability pension, work assessment allowance, and more symptoms of anxiety and depression were associated with lower EQ-5D index and EQ VAS scores. Furthermore, being male, use of pain medication and having disorders related to trauma were associated with reduced EQ-5D index scores, while hyperkinetic disorders were associated with higher EQ-5D index scores.

**Conclusion:**

HRQoL of psychiatric outpatients is clearly impaired. This study indicated a significant association between employment status, symptom severity, and HRQoL in treatment-seeking outpatients. The findings highlight the importance of assessing HRQoL as part of routine clinical assessment.

**Supplementary Information:**

The online version contains supplementary material available at 10.1007/s11136-024-03748-3.

## Introduction

Health-related quality of life (HRQoL) serves as a comprehensive indicator of a person’s well-being in mental and physical aspects related to level of daily functioning [1]. Globally, persons with common mental disorders (CMD) account for one third of years lived with disability [2] and their HRQoL is reduced compared to healthy individuals [3–5]. For this reason, increased awareness has been devoted to assessing HRQoL in addition to measures of symptom severity [6] in mental health care.

Early studies on quality-of-life (QoL) in people with anxiety and depression found that QoL was significantly reduced and could vary according to diagnoses. Proportion of patients with a clinically severe QoL impairment ranged from 63% for depression, 59% for post-traumatic stress disorder (PTSD), 26% for obsessive-compulsive disorder (OCD), to 21% for social phobia [7]. These early studies typically used the Short Form Health Survey to assess quality of life, often finding most pronounced effects on the social functioning and mental health components, but also for work, physical health, and home and family [8]. A more recent review found that QoL is reduced before disorder onset, drops during the disorder, and improves following treatment as changes in anxiety and depression are associated with changes in QoL [9]. QoL relates to satisfaction with one’s life, while HRQoL could be defined as the way health affects QoL [10]. For HRQoL, common mental health problems are associated with lower HRQoL compared to people with common medical disorders such as back/neck problems, diabetes, and hypertension [11]. This corresponds with research on quality-adjusted life-years showing that the greatest impact was from arthritis, mood disorders, and personality disorders [12].

The association between mental illness and HRQoL varies across disorders. Of those disorders frequently encountered in outpatient psychiatric settings, anxiety disorders and depressive disorders have reduced HRQoL [8, 13]. The same is true for patients with PTSD [14, 15], who also have been reported to have lower HRQoL than other anxiety disorders [16]. Patients with personality disorders report lower HRQoL compared to patients with other mental disorders [17–19] and to have several personality disorders is associated with even lower EQ-5D values [20, 21]. On the other hand, patients with attention-deficit and hyperactivity disorder (ADHD), have been found to have lower HRQoL than the general population, but better HRQoL compared to depression and personality disorders [22, 23], and comorbidity has been associated with lower HRQoL [24, 25].

The Norwegian mental healthcare is structured with two main levels: primary care and specialist care. Primary care is usually the first level of care and patients are referred to specialist health care if more specialised treatment is needed, for instance due to a higher severity level. Factors associated with HRQoL in the Norwegian primary mental healthcare have been investigated [26], but these results may not be applicable to patients in specialist care, as the latter are expected to have higher symptom severity and more functional impairment, and thus lower HRQoL. Previous research within Norwegian specialist healthcare restricted to a limited number of mental disorders like anxiety and depression may not be generalizable to more heterogeneous patient samples [27]. For instance, personality disorders are found to be a substantial subgroup of patients in psychiatric outpatient care [28]. There is a need for studies investigating HRQoL scores across different types of patient groups frequently encountered in routine specialist mental healthcare.

Studies have found women to report lower HRQoL [29, 30], while other studies have not found gender differences [26]. Clinical variables like symptoms of anxiety and depression, comorbidity, more subjective health complaints and work impairment or sick leave have been associated with lower HRQoL [27, 31, 32]. Further, psychotropic medication is often prescribed to patients with mental disorders, and use of antidepressants and pain medication have been found related to lower HRQoL [33, 34].

Many of the previous studies were disorder specific, and the reported HRQoL may not be generalizable to psychiatric outpatient samples that typically are very heterogeneous. There is therefore a need for studies investigating HRQoL and factors related to HRQoL in heterogeneous patient populations referred for treatment in specialist healthcare. Moreover, research using the EuroQoL 5-Dimension 5-level (EQ-5D-5L) to assess HRQoL in heterogeneous samples with common mental health disorders is lacking.

In this study, we aimed to investigate factors associated with HRQoL by applying the EQ-5D-5L in a large and heterogeneous patient sample referred for treatment in specialist mental healthcare. Further, we aimed to study differences in HRQoL across mental disorders. In accordance with previous studies, we anticipated that all patient groups would score lower on HRQoL than the general population. Personality disorders were expected to report lower HRQoL than patients with anxiety disorders, depressive disorders, PTSD and trauma-related disorders, and ADHD.

## Methods

### Sample and procedures

This study reports on data from an observational routine treatment monitoring study with a cross-sectional design. Patients at a psychiatric outpatient facility in the specialist healthcare were invited to participate. In the Norwegian healthcare system, patients must have a mental condition that requires more specialised mental healthcare than provided in the municipality healthcare to be entitled specialist health care, and they must be referred by their general practitioner. As part of routine assessment patients were diagnosed by a clinician using the Mini International Neuropsychiatric Interview [35]. Diagnoses were clustered within their overarching block of the ICD-10 chapter V Mental and behavioural disorders (F00-F99) [36]. We included the following groups of disorders: F30 (Mood disorders), F40 (Neurotic, stress-related and somatoform disorders), F60 (Disorders of adult personality and behaviour), and F90 (Behavioural and emotional disorders with onset usually occurring in childhood and adolescence). These were labelled depressive-, anxiety, personality and hyperkinetic disorders, respectively. In addition, patients diagnosed within F43 Adjustment disorders and F44 Dissociative disorders were labelled “trauma-related disorders” and were treated as a separate diagnostic group from the remaining F40 disorders, corresponding to the DSM-5 categorisation, where trauma- and stressor-related disorders and anxiety disorders are separated. We did not have access to information regarding chronicity of the disorders. The period of data collection was from March 12, 2020, to November 7, 2023.

**Table 1:** Background characteristics of sample participants (*N* = 1947)

	Mean (SD)
EQ-5D-5L	
EQ-5D index	0.46 (0.25)
EQ-5D VAS Health 0-100	47.45 (18.79)
Age	32.20 (10.25)
PHQ-9	16.05 (5.51)
GAD-7	12.43 (4.75)
PHQ-ADS	28.49 (9.14)
	Frequency (%)
Sex	
Female	1262 (64.88)
Male	685 (35.18)
Married/cohabitant/in a relationship	799 (41.04)
Occupation status	
Working/studying	1056 (54.24)
Sick leave	546 (28.04)
Disability benefits	80 (4.11)
WAA	265 (13.61)
Antidepressants	321 (16.49)
Anxiolytics	40 (2.05)
Pain medication	28 (1.44)
Hypnotics	25 (1.28)
Psychostimulants	77 (3.96)
Primary disorders	
F30 Depressive	741 (38.06)
F40 Anxiety	481 (24.70)
F60 Personality	194 (9.96)
F90 Hyperkinetic	315 (16.18)
Trauma-related	216 (11.09)
Comorbidity	630 (32.40)
Comorbid disorders	
F10 Substance use	14 (0.70)
F30 Depressive	250 (12.84)
F40 Anxiety	249 (12.79)
F50 Eating	28 (1.43)
F60 Personality	3 (0.15)
F80 Development	19 (0.97)
F90 Hyperkinetic	65 (3.34)
Trauma-related	2 (0.10)

Note. VAS = Visual Analogue Scale. PHQ-9 = Patient Health Questionnaire 9. GAD-7 = Generalized Anxiety Disorder 7. PHQ-ADS = Patient Health Questionnaire Anxiety and Depression Scale. WAA = Work Assessment Allowance

### Measures

As part of routine screening procedures, respondents self-reported if they were currently working/studying, if they recently had been or currently were on sick leave, current use of psychotropic medication, and relationship status, which was coded as being in a relationship or being single. Information regarding age, sex and psychiatric diagnoses were retrieved from clinical register data. Data on disability benefits and Work Assessment Allowance (WAA) were extracted from the Norwegian Labour and Welfare Administration (NAV). Self-report questionnaires were completed before treatment through an online survey portal (CheckWare), which applies electronic ID for secure login. Respondents received an SMS with a link to the survey, and they received a reminder if the survey was not completed within a week.

EQ-5D-5L [37] is a five-item questionnaire that measures HRQoL. This is a preference-based measure, i.e. the value of different health states is rated according to an individual’s preferences, which has frequently been used to assess HRQoL [38]. It contains five dimensions (mobility, self-care, usual activities, pain/discomfort, and anxiety/depression) and respondents rate their perceived health on each domain (e.g. mobility) by choosing from five response labels on a level of severity from 1 (no problems; *I have no problems in walking about*) to 5 (severe problems; *I am unable to walk about*). The score from each item is combined into a five-digit EQ-5D-5L profile. By use of country-specific preference weights, the profile may be calculated to an EQ-5D index score from 0 (death) to 1 (perfect health). In general population studies, the EQ-5D index value is reported to range from 0.81 to 0.87 [3, 39–41]. As country-specific weights do not exist for Norway a “crosswalk” technique was applied using UK preference weights [42].

The EQ-5D-5L also includes a visual analogue scale which asks the respondents “We would like to know how good or bad your health is today”, which is scored on a scale from 0 (*The worst health you can imagine*) to 100 (*The best health you can imagine*).

The EQ-5D-5L has previously been used to study HRQoL in patients with CMD in specialist health care, demonstrating good psychometric properties [22, 43]. In Norway the scale has been validated in patients with depression and anxiety at risk of sick leave [27, 44] as well as in the general population [39]. For mental health populations, the EQ-5D-5L has good psychometric properties in patients with depression, anxiety and personality disorders [45–47]. Internal reliability was not expected to be high as the EQ-5D-5L measures five separate domains [48]. Cronbach’s alpha in the current study was 0.69.

The Patient Health Questionnaire 9 (PHQ-9) [49] was used to measure symptoms of depression. The PHQ-9 has nine items which are rated on a four-point Likert scale from 0 (not at all) to 3 (nearly every day). The total score range is from 0 to 27, and a higher score indicates more severe depressive symptoms. The psychometric properties of the PHQ-9 are well-established, including for the Norwegian translation [50]. Internal reliability in this study was good, with a Cronbach’s alpha of 0.82.

The Generalized Anxiety Disorder 7 (GAD-7) [51] was included to measure symptoms of anxiety. The GAD-7 measures the severity of symptoms with seven items that are scored on a scale from 0 (not at all) to 3 (nearly every day). The total score ranges from 0 to 21, with higher scores indicating higher severity. Although the GAD-7 was originally developed to screen for symptoms of generalized anxiety disorder, studies show that the instrument also is a useful screener of general anxiety symptoms in heterogeneous patient samples [52]. The internal reliability was good in this study (Cronbach’s alpha = 0.84).

The PHQ-9 and GAD-7 were combined to form the Patient Health Questionnaire Anxiety and Depression Scale (PHQ-ADS) [53], which has a total score range from 0 to 48. The PHQ-ADS has good psychometric properties [54]. The Cronbach’s alpha for the 16-item PHQ-ADS was 0.88.

### Statistical analysis

All analyses were conducted in the statistical environment of R. Little’s Missing Completely at Random (MCAR) test was used to identify missing data patterns, with the null hypothesis that data is missing completely at random. The analysis indicated that data was missing completely at random. To make use of all available data, replacement of missing values was handled by multiple imputation (MI), as MI is argued to reduce bias compared to complete case analysis [55–57]. MI by chained equations was conducted using the MICE package [58].

Differences between groups of mental disorders on the EQ-5D index and VAS were tested with ANOVAs, with Games-Howell corrected post-hoc tests. Partial eta squared was calculated as effect size and an effect size of 0.01 was interpreted as small, 0.06 as medium and 0.14 as large [59]. Two separate stepwise regression analyses were conducted with EQ-5D index and EQ VAS as outcomes. The first step controlled for background variables (age, sex, and relationship status). The second step controlled for occupational status, with working/studying as reference. In the third step, the use of anxiolytics, antidepressants, pain medication, hypnotics and psychostimulants were included as separate binary variables (yes or no). In the fourth step, PHQ-ADS was included. In the final step, comorbidity and diagnostic groups were added, with the subgroup F30 depressive disorders as reference.

#### Power analysis

To identify small effect sizes, with α = 0.05 and power = 0.80, a required sample size of *N* = 1200 would be needed for the ANOVAs (5 groups), and *N* = 878 for the regression models (12 predictors). The sample size was therefore acceptable.

#### Sensitivity analysis

Initial screening of the data showed skewed values for the EQ-5D index and EQ VAS, and the Breusch-Pagan test indicated an issue with heteroscedasticity for the regression models. We, therefore, repeated the regression analyses with robust standard errors [60] using the *lm_robust* function from the *estimatr* package [61]. In addition, the regression analysis with the EQ-5D index as outcome was repeated where the PHQ-ADS variable was omitted as a covariate.

## Results

Patients were invited to participate by an SMS that included a link directing them to the online consent form and self-report questionnaires. A total of 3789 responded to the invitation link, of whom 3584 consented to participate and 205 declined. Of those who consented, clinical variables from patient registers were available for 3201 patients. Patients with other disorders (*n* = 1190), and participants who had not completed the EQ-5D-5L (*n* = 64), were removed. Data from a total of 1947 respondents (65% female) with a mean age of 32.2 years (*SD* = 10.3) were included in the analyses. The sample reported a mean PHQ-9 score of 16.1 (*SD* = 5.51), which indicates moderate-severe symptoms of depression. Except for patients with anxiety disorders who reported depressive symptoms of moderate severity, all diagnostic subgroups reported moderately severe depressive symptoms (PHQ-9 range 15–19) [49]. The mean GAD-7 score was 12.4 (*SD* = 4.75) which indicates moderate symptoms of generalized anxiety. All diagnostic subgroups reported generalized anxiety symptoms in the moderate severity range (GAD-7 range 10–14) [51]. See Table 1 for background characteristics of the sample. Detailed diagnostic information is provided in Table S1 in the Supplementary Material.

In the dataset, 16 (0.8%) had one or more items missing on the EQ-5D-5L, 11 (0.6%) had missing on the EQ VAS, 142 (7.3%) respondents had not reported their relationship status, and five (0.3%) had missing values on PHQ-ADS. There were no missing values on the remaining variables.

### Distribution of EQ-5D-5L domains

Table 2 displays reported problems across EQ-5D-5L domains for patients with single diagnoses and comorbid patients. The proportion reporting problems among the non-comorbid diagnostic subgroups was overall lower for patients with hyperkinetic disorders than the other diagnoses. Those with personality disorders reported the highest proportion of problems in all domains except for pain/discomfort and self-care, where patients with comorbid conditions reported more problems.

**Table 2:** EQ-5D-5L domains, EQ-5D index, EQ VAS and symptom severity by non-comorbid diagnostic subgroups and comorbid disorders

	Depressive (*n* = 552)	Anxiety (*n* = 310)	Personality (*n* = 87)	Hyperkinetic (*n* = 223)	Trauma (*n* = 145)	Comorbid (*n* = 630)
Mobility (%)						
No problems	302 (54.7)	184 (59.4)	38 (43.7)	138 (61.9)	72 (49.7)	288 (45.7)
Slight problems	132 (23.9)	64 (20.6)	26 (29.9)	47 (21.1)	28 (19.3)	166 (26.3)
Moderate problems	82 (14.9)	42 (13.5)	14 (16.1)	28 (12.6)	30 (20.7)	124 (19.7)
Severe problems	34 (6.2)	16 (5.2)	8 (9.2)	9 (4.0)	12 (8.3)	50 (7.9)
Unable to do	2 (0.4)	4 (1.3)	1 (1.1)	1 (0.4)	3 (2.0)	2 (0.3)
Proportion reporting problems	250 (45.3)	126 (40.7)	49 (56.3)	85 (38.1)	73 (50.4)	342 (54.3)
Self-care (%)						
No problems	47 (8.5)	39 (12.6)	7 (8.1)	26 (11.7)	16 (11.1)	42 (6.7)
Slight problems	105 (19.0)	107 (34.5)	24 (27.6)	63 (28.3)	46 (31.9)	130 (20.7)
Moderate problems	207 (37.5)	97 (31.3)	27 (31.0)	72 (32.3)	39 (27.1)	223 (35.5)
Severe problems	174 (31.5)	65 (21.0)	23 (26.4)	55 (24.7)	39 (27.1)	210 (33.4)
Unable to do	19 (3.4)	2 (0.6)	6 (6.9)	7 (3.1)	4 (2.8)	23 (3.7)
Proportion reporting problems	505 (91.5)	271 (87.4)	80 (92.0)	197 (88.3)	128 (88.3)	586 (93.0)
Usual activities (%)						
No problems	340 (61.8)	229 (73.9)	47 (54.6)	164 (73.9)	105 (72.4)	367 (58.4)
Slight problems	129 (23.5)	58 (18.7)	20 (23.3)	37 (16.7)	29 (20.0)	169 (26.9)
Moderate problems	67 (12.2)	16 (5.2)	17 (19.8)	17 (7.6)	6 (4.1)	73 (11.6)
Severe problems	14 (2.5)	6 (1.9)	2 (2.3)	4 (1.8)	5 (3.4)	19 (3.0)
Unable to do	0 (0.0)	1 (0.3)	0 (0.0)	0 (0.0)	0 (0.0)	0 (0.0)
Proportion reporting problems	210 (38.0)	81 (26.1)	39 (44.8)	58 (26.0)	40 (27.6)	261 (41.4)
Pain/discomfort (%)						
No problems	112 (20.3)	67 (21.6)	19 (21.8)	68 (30.8)	15 (10.4)	114 (18.1)
Slight problems	232 (42.0)	111 (35.8)	26 (29.9)	77 (34.8)	37 (25.7)	215 (34.2)
Moderate problems	135 (24.5)	85 (27.4)	23 (26.4)	52 (23.5)	52 (36.1)	181 (28.8)
Severe problems	57 (10.3)	39 (12.6)	15 (17.2)	17 (7.7)	31 (21.5)	90 (14.3)
Extreme	16 (2.9)	8 (2.6)	4 (4.6)	7 (3.2)	9 (6.3)	29 (4.6)
Proportion reporting problems	440 (79.7)	243 (78.4)	68 (78.2)	153 (68.6)	129 (89.0)	515 (81.8)
Anxiety/depression (%)						
No problems	23 (4.2)	13 (4.2)	3 (3.4)	38 (17.0)	10 (7.1)	20 (3.2)
Slight problems	76 (13.8)	57 (18.4)	12 (13.8)	75 (33.6)	28 (19.9)	74 (11.8)
Moderate problems	206 (37.4)	127 (41.0)	28 (32.2)	69 (30.9)	54 (38.3)	216 (34.4)
Severe problems	216 (39.2)	98 (31.6)	36 (41.4)	33 (14.8)	41 (29.1)	260 (41.4)
Extreme	30 (5.4)	15 (4.8)	8 (9.2)	8 (3.6)	8 (5.6)	58 (9.2)
Proportion reporting problems	528 (96.0)	297 (95.6)	84 (97.0)	185 (83.0)	131 (90.4)	608 (96.5)
EQ VAS (mean (SD))	46.5 (18.3)	50.7 (18.5)	43.5 (18.9)	54.1 (20.2)	45.1 (18.9)	45.4 (18.1)
EQ-5D index (mean (SD))	0.47 (0.24)	0.51 (0.24)	0.41 (0.27)	0.57 (0.24)	0.43 (0.26)	0.42 (0.25)
PHQ-9 (mean (SD))	16.9 (5.1)	13.4 (5.3)	17.3 (5.9)	14.1 (5.7)	15.2 (5.5)	17.33 (5.2)
GAD-7 (mean (SD))	11.8 (4.7)	12.3 (4.6)	13.3 (5.0)	11.0 (4.9)	12.3 (4.6)	13.5 (4.6)

Note. Diagnostic subgroups include only single-disorder cases. Trauma = Trauma-related disorders. Data are displayed before multiple imputation, hence numbers may not add up to totals due to missing responses

### EQ-5D index

For the complete sample the mean EQ-5D index was 0.46 (*SD* = 0.25). Those with a diagnosis of a personality disorder and trauma-related disorders reported the lowest EQ-5D index, both with a mean score of 0.40 (*SD* = 0.26), followed by depressive (*M* = 0.46, *SD* = 0.24) and anxiety disorders (*M* = 0.49, *SD* = 0.24). Those with hyperkinetic disorders reported the highest EQ-5D index (*M* = 0.53, *SD* = 0.25). The difference in values was statistically significant (*F*(4) = 15.07, *p* < 0.001, *ηρ²* = 0.03). Games-Howell corrected post-hoc tests showed that both personality and trauma-related disorders scored significantly lower than depressive, anxiety and hyperkinetic disorders.

### EQ VAS

The complete sample reported a mean EQ VAS value of 47.45 (SD = 18.79). Those with a personality disorder diagnosis reported the lowest EQ VAS with a mean score of 43.70 (*SD* = 18.30), followed by trauma-related disorders (*M* = 44.50, *SD* = 19.00), depressive disorders (*M* = 46.30, *SD* = 18.30) and anxiety disorders (*M* = 49.40, *SD* = 18.30). Those with hyperkinetic disorders reported the highest EQ VAS score (*M* = 51.60, *SD* = 20.00). The differences between the diagnostic subgroups were statistically significant (*F*(4) = 9.328, *p* < 0.001, *ηρ²* = 0.02). Games-Howell adjusted post-hoc tests showed that personality scored significantly lower than anxiety and hyperkinetic disorders, trauma-related scored significantly lower than anxiety and hyperkinetic disorder, depressive scored significantly lower than anxiety disorders, and hyperkinetic scored significantly higher than depressive disorders.

### EQ-5D index and EQ VAS - comparisons with primary care and the general population

**Fig. 1:**
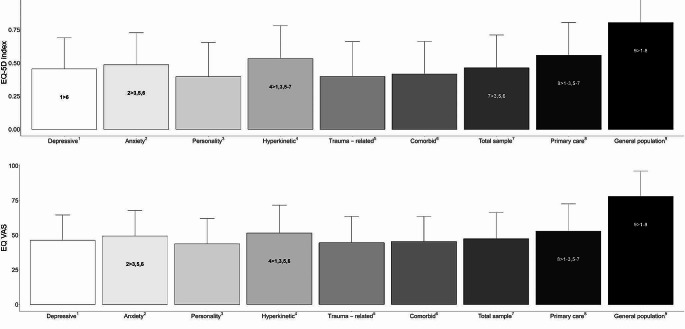
Comparisons between diagnostic subgroups and total sample with primary care and the general population for EQ-5D index and EQ VAS. Note: Annotated subgroup differences are based on Games-Howell corrected post-hoc tests. Primary care [34]. General population [39]

Both the diagnostic subgroups and the total sample scored lower on both the EQ-5D index and the EQ VAS than the general population [39] and Norwegian primary mental health services [34]. For the EQ-5D index, there were statistically significant differences between the total sample and the primary care sample, (*mean difference* [*MD*] = 0.10, 95% *CI* = 0.06–0.13, *p* < 0.001, Cohen’s *d* = 0.39), and the general population (*MD* = 0.34, 95% *CI* = 0.31–0.37, *p* < 0.001, Cohen’s *d* = 1.52). For the EQ VAS, there were statistically significant differences between the total sample and the primary care sample (*MD* = 5.45, 95% *CI* = 2.75–8.15, *p* < 0.001, Cohen’s *d* = 0.28) and the general population (*MD* = 30.4, 95% *CI* = 27.9–33.0 *p* < 0.001, Cohen’s *d* = 1.64). Figure 1 shows comparisons between the diagnostic subgroups and the total sample with the general population and primary care.

### Variables associated with the EQ-5D index

The stepwise linear regression analyses with the EQ-5D index as outcome (see Table 3), male gender, being on sick leave, being on disability pension, receiving WAA, a higher score on the PHQ-ADS, use of pain medication, and trauma-related disorders were associated with lower EQ-5D index. In contrast, hyperkinetic disorders were associated with higher EQ-5D index. Symptoms of anxiety and depression showed the strongest association with the EQ-5D index. The model explained 40% (adjusted *R*^*2*^) of the variance.

**Table 3:** Regression analysis with EQ-5D index as an outcome using multiple imputation

Step	Variable	Adjusted *R*^2^	F change
1	*Demographics*	0.01	
2	*Work*	0.05	33.001*
3	*Medication*	0.06	5.840*
4	*Symptoms*	0.40	1078.272*
5	*Comorbidity*	0.40	2.174
6	*Diagnoses*	0.40	6.043*

**Variable**	***B***	**95% CI**	***p*** **-value**
Age	0.00	0.00, 0.00	0.700
Male sex	-0.02	-0.04, 0.00	0.013
In a relationship	0.01	-0.01, 0.03	0.500
Occupation status^1^			
Sick leave	-0.03	-0.06, -0.01	0.002
Disability pension	-0.12	-0.17, -0.07	< 0.001
WAA	-0.09	-0.12, -0.07	< 0.001
Antidepressants	-0.01	-0.03, 0.01	0.400
Anxiolytics	-0.05	-0.11, 0.01	0.090
Pain medication	-0.09	-0.16, -0.01	0.020
Psychostimulants	-0.01	-0.06, 0.03	0.600
Hypnotics	-0.02	-0.09, 0.06	0.700
PHQ-ADS	-0.02	-0.02, -0.01	< 0.001
Comorbidity	-0.01	-0.03, 0.01	0.300
Diagnoses^2^			
Anxiety	-0.01	-0.03, 0.01	0.400
Personality	-0.03	-0.06, 0.01	0.100
Hyperkinetic	0.03	0.00, 0.06	0.020
Trauma-related	-0.05	-0.08, -0.02	0.001

Note. *B* = unstandardized coefficient.CI = Confidence Interval. ^1^ Reference = Working or studying. ^2^ Reference = Depressive disorders. * *p* < 0.001. PHQ-ADS = Patient Health Questionnaire Anxiety and Depression Scale. WAA = Work Assessment Allowance

As sensitivity analyses, the regression analysis was repeated with robust standard errors. All results were identical to the main analysis, except for use of pain medication which was no longer significant. Moreover, we repeated the regression analysis with the EQ-5D index as outcome, with the PHQ-ADS excluded as covariate. In the additional analysis, relationship status, anxiety medication, comorbidity, and anxiety disorders turned significant. Male gender was no longer a significant covariate. Sick leave, disability benefits, WAA, pain medication, hyperkinetic and trauma-related disorders remained significant covariates. The total variance explained was 9%.

### Variables associated with the EQ VAS

In the regression analysis with the EQ VAS as outcome (see Table 4), the results showed that being on sick leave, disability pension, receiving WAA, and a higher score on PHQ-ADS were associated with a lower score on the EQ VAS. The variance explained (adjusted *R*^*2*^) for the model was 26%, where symptoms of anxiety and depression accounted for most of the variance explained. The sensitivity analysis with robust standard errors showed equal results as the main analysis.

**Table 4:** Regression analysis with EQ VAS as an outcome using multiple imputation

Step	Variable	Adjusted *R*^2^	F change
1	*Demographics*	0.01	
2	*Work*	0.06	33.504*
3	*Medication*	0.07	4.186*
4	*Symptoms*	0.26	498.385*
5	*Comorbidity*	0.26	0.044
6	*Diagnoses*	0.26	1.288

**Variable**	***B***	**95% CI**	***p*** **-value**
Age	-0.08	-0.16, 0.01	0.072
Male sex	-0.77	-2.30, 0.79	0.300
In a relationship	0.34	-1.20, 1.90	0.700
Occupation status^1^			
Sick leave	-4.20	-6.00, -2.40	< 0.001
Disability pension	-5.80	-9.80, -1.80	0.004
WAA	-8.50	-11.00, -6.20	< 0.001
Antidepressants	-1.50	-3.40, 0.52	0.150
Anxiolytics	0.63	-4.60, 5.80	0.800
Pain medication	-2.30	-8.40, 3.80	0.500
Psychostimulants	3.60	-0.40, 7.50	0.078
Hypnotics	4.00	-2.50, 11.00	0.200
PHQ-ADS	-0.91	-1.00, -0.83	< 0.001
Comorbidity	0.31	-1.30, 1.90	0.700
Diagnoses^2^			
Anxiety	0.33	-1.60, 2.20	0.700
Personality	-1.30	-3.90, 1.40	0.300
Hyperkinetic	1.50	-0.74, 3.80	0.200
Trauma-related	-1.30	-3.80, 1.20	0.300

Note. *B* = unstandardized coefficient. CI = Confidence Interval. ^1^ Reference = Working or studying. ^2^ Reference = Depressive disorders. * *p* < 0.001. PHQ-ADS = Patient Health Questionnaire Anxiety and Depression Scale. WAA = Work Assessment Allowance

## Discussion

This study aimed to explore HRQoL in outpatients with CMD seeking treatment at a specialist mental health care clinic. The sample included patients with anxiety, depressive, personality, hyperkinetic and trauma-related disorders. Overall, we found that patients with personality and trauma-related disorders reported the lowest scores on the EQ-5D index and EQ VAS, followed by depression and anxiety, with hyperkinetic disorders reporting the highest scores. The results are expected when compared to previous studies of HRQoL using the EQ-5D-5L [22, 27, 47, 62] and support the scale as a useful measure of HRQoL also in heterogeneous psychiatric outpatient samples.

The complete sample reported an EQ-5D index of 0.46, and an EQ VAS score of 47.5. The sample scored lower than the general population and patients in the Norwegian primary mental health service [34, 39]. The Norwegian healthcare system is organised in such a way that patients in need of more specialised care are referred from primary care to specialist care, which may explain the lower scores in our study. The EQ-5D index and EQ VAS scores are also lower than a previous sample treated in the Norwegian specialist health care [63] which included patients referred for psychotherapy and work-focused interventions due to anxiety and/or depression. Another Norwegian study in the specialist health care [32] reported considerably higher EQ VAS scores on the EQ-5D-3 L for patients with CMD, compared to our study and Lindberg et al. [34]. However, in the study by Reme et al. [32] CMD was evaluated based on self-report questionnaires, while we extracted diagnoses from medical records based on clinician-administered structured interviews, and the difference in diagnostic procedures may contribute to explain the differences in HRQoL scores. Moreover, the sample in the current study is characterized by great heterogeneity and included several mental disorders that are frequently treated in specialist healthcare. This may explain the more severe HRQoL compared to what has been reported in several previous studies. The present study adds to the literature by reporting HRQoL in a representative sample of psychiatric outpatients and provides a more accurate description of routine clinical settings where a broad range of mental disorders are frequently encountered.

The overall EQ VAS score in the current study was higher than what has been reported in psychiatric samples in England and the Netherlands [22], but similar to a Spanish study within the specialist health service [43]. However, in the latter study, the reported EQ-5D index was higher compared to our results. The EQ-5D index is derived from five different domains and calculated based on country specific weights, whereas the EQ VAS is based on one question which is not weighted. This may contribute to explain the greater similarity in EQ VAS scores compared to EQ-5D index scores. Additionally, possible cultural differences have been suggested to influence HRQoL, for example, the Spanish culture has been described more collectivistic compared to northern European countries [64]. Furthermore, previous studies have shown that immigrants representing an ethnic minority or originating from countries with societal challenges report reduced HRQoL [26, 65, 66]. However, the potential cultural impact on HRQoL is debated, and although health utilities are shown to vary between countries, cultural values have not systematically been shown to explain these differences [67]. Instead, country-specific weighting has been recommended, and the results of this study should therefore be replicated in the future when Norwegian value sets are available.

The reduced EQ-5D index and EQ VAS scores in patients with depressive and anxiety disorders were comparable to previous research [9]. Studies show that depression has high comorbidity with other mental disorders [68], and e.g. comorbid depression and personality disorders have been associated with worse HRQoL [69]. Our results showed that the EQ-5D index and EQ VAS were lower in comorbid patients than patients with single disorders, except for personality disorders. However, in routine clinics screening for comorbidity, including personality disorders, is often not conducted in a systematic manner [28, 70], perhaps due to high caseloads among clinicians, and it is possible that the degree of comorbidity is underreported in this study.

The EQ-5D index score reported by patients with personality disorders were, with the exception of trauma-related disorders, lower than the remaining diagnostic subgroups in the current study, which supports previous studies where personality disorders were systematically associated with lower HRQoL [12, 20, 21]. Personality disorders are found to be frequent, yet often undiagnosed, in specialist healthcare [28, 70, 71]. It is a strength that such disorders are included in the current study, as this reflects the heterogeneity of psychiatric outpatients more accurately. However, in Norwegian routine clinical settings a practice of underdiagnosing personality disorders has been suggested [72]. This may thus be true for the current sample as well, meaning that the actual prevalence of personality disorders is higher than the numbers reported. In recent years, effective treatments have been developed for several personality disorders, and a clinical implication of our study is that health professionals should strengthen the recognition of personality disorders as part of the routine assessment, to make effective treatment available for patients with personality disorders to improve HRQoL in this patient group.

The overall reduced HRQoL reported by patients with trauma-related disorders corroborates previous studies [8, 73]. In the DSM-IV and the ICD-10, trauma-related disorders are categorised as anxiety disorders. However, in this study we treated trauma-related disorders, including PTSD, as a separate subgroup from anxiety disorders. This categorisation is in line with the DSM-5 which included a separate chapter for Trauma and Stressor-Related Disorders. A possible explanation for the low HRQoL reported by patients with trauma-related disorders is that the patients had suffered multiple traumas. Furthermore, previous research has noted a high symptom overlap between complex PTSD and personality disorders [74] and a comorbidity rate of up to 50% between the two disorders [75]. Considering the aforementioned fact that comorbidity and personality disorders are underdiagnosed in routine clinics, the association between trauma-related disorders, personality disorders and HRQoL should be investigated further in future studies.

Patients with hyperkinetic disorders reported the highest EQ-5D index. The index value is lower than a Swedish study for newly diagnosed patients with ADHD [23] and similar to a Swedish study of unremitted ADHD patients [76]. Combined with an increased awareness that ADHD persists into adulthood [77] and increased use of psychostimulants in the Nordic countries [78], there has been an increase in referrals to the specialist healthcare for assessments of hyperkinetic disorders. The higher HRQoL reported by patients with hyperkinetic disorders in this study indicates that, on the group level, these patients have less functional impairment than the other disorders studied. This finding may inform health policy decisions, as the allocation of resources is often based on an evaluation of patients’ impairment, their need of care and the expected benefit from the care provided.

Being on sick leave, disability pension, WAA, and higher symptom level were associated with lower EQ-5D index and EQ VAS scores, which is in line with previous Norwegian and international studies [27, 34, 79]. These findings have clinical relevance and suggest that mental health treatment should aim at increasing work participation, as treatment interventions aimed at both symptom reduction and improved work functioning are beneficial for patients with mental disorders [80].

Moreover, we found that patients with trauma-related disorders and men were associated with lower scores on the EQ-5D index, but not the EQ VAS. The former finding corroborates previous studies that found anxiety disorders and PTSD to have reduced HRQoL [8]. The latter finding contradicts previous research which has found women to report lower EQ-5D index scores than men [27, 81], or no gender differences [34].

Pain medication was associated with lower EQ-5D index, which corroborated a Norwegian study in primary health care [34]. This finding is noteworthy, as it may be expected that there are more patients with a need for pain medication treated in primary care. The underlying pathology causing a need for pain medication is likely to impact HRQoL to a great extent, however, we did not have access to medical records to study this in detail. Mental health professionals should routinely screen for symptoms of pain, as the co-occurrence of pain and mental health problems may require specifically tailored interventions [82]. However, the finding must be treated with caution, as pain medication was not a significant covariate when robust regression analysis was applied. The results did not reveal significant associations between other psychotropic drugs and HRQoL, however, we did not explore if the association between medication use and HRQoL varied between diagnoses, which may be a relevant area for future research, as clinical guidelines recommend different medications to be prescribed for various mental disorders.

Symptoms of anxiety and depression were most strongly related to the EQ-5D index and the EQ VAS. This was expected, as previous studies have found higher symptom levels to be associated with lower scores on the EQ-5D index and the EQ VAS [27, 83]. However, researchers have warned about a potential overlap between symptom measures and measures of HRQoL [84]. The index value is derived from the five EQ-5D domains, which include anxiety/depression. We therefore conducted an additional analysis excluding the measure of anxiety and depression symptoms. In the analysis, being in a relationship and anxiety disorders were associated with better HRQoL, and use of anxiety medication and comorbidity were associated with worse HRQoL. The association between gender and HRQoL ceased to be significant, while the remaining variables had equal effects as in the original analysis. This analysis explained a smaller amount of the variance in the EQ-5D index compared to the analysis that included the symptom measure. The fact that psychological symptoms had such a profound effect on the outcome, suggests that future studies should be careful when considering controlling for symptoms of anxiety and depression, due to the potential overlap with HRQoL.

### Limitations

A strength of this study is the large heterogeneous sample with clinician-assessed mental disorders representative of outpatient specialist mental health care. However, there are limitations that should be noted. The cross-sectional design limits the conclusion that can be drawn, and future studies should include post-treatment assessment to evaluate factors related to change in HRQoL. The use of psychotropic medication was self-reported and not retrieved from registers at the treatment facility. Moreover, medication use was recorded at the time of referral, and medication dosage may have been changed or discontinued after treatment start. Substance consumption was not reported, which is a limitation given that previous studies have shown a high comorbidity rate between mental disorders and substance use disorder [85], and further, that dual diagnoses are associated with lower HRQoL [86, 87]. Patients with psychotic disorders were treated at different units than those included in this study, which is a limitation, and consequently the findings of the current study may not generalize to more heterogeneous samples that include patients diagnosed with psychotic disorders. Furthermore, the Cronbach’s alpha of the EQ-5D-5L was below the recommended cut-off of 0.70 [88]. However, it has been argued that internal consistency is not a good indication of psychometric properties for the EQ-5D-5L, as it is a multidimensional construct based on five domains that are separate from each other. Hence, internal reliability is not expected to be high [48, 89] and previous research has reported equally low internal reliability [90]. Lastly, the EQ-5D-5L is limited to five domains, and future studies may consider using alternative measures, such as the SF-36 [91], to capture other aspects of relevance for HRQoL.

## Conclusion

The present study indicates that treatment seeking patients in specialist mental healthcare are characterised by reduced HRQoL compared both to the general population and primary care patients. The factors most strongly related to HRQoL were occupational status and mental health symptoms, which suggests that both are important areas to target in clinical work. The study shows the importance of routinely assessing HRQoL in patients with CMD.

## Electronic supplementary material

Below is the link to the electronic supplementary material.

Supplementary Material 1

Supplementary Material 2

## Data Availability

Data are available from the corresponding author upon reasonable request.
