# Supplementary material for: Health-related quality of life in psychiatric outpatients: a cross-sectional study of associations with symptoms, diagnoses, and employment status
Source: Qual Life Res. 2024 Aug 7;33(11):3093–105. doi: 10.1007/s11136-024-03748-3 (PMC11541330; doi:10.1007/s11136-024-03748-3)
Supplement: Supplementary file 1 — Supplementary Material 1 [file 11136_2024_3748_MOESM1_ESM.docx]

STROBE Statement—checklist of items that should be included in reports of observational studies

|  | Item No. | Recommendation | Page  No. | Relevant text from manuscript |
| --- | --- | --- | --- | --- |
| **Title and abstract** | 1 | (*a*) Indicate the study’s design with a commonly used term in the title or the abstract | 1 | Health-related quality of life in psychiatric outpatients: A cross-sectional study of associations with symptoms, diagnoses, and employment status |
|  |  | (*b*) Provide in the abstract an informative and balanced summary of what was done and what was found | 2 | Abstract |
| Introduction | | | |  |
| Background/rationale | 2 | Explain the scientific background and rationale for the investigation being reported | 2 | See introduction section |
| Objectives | 3 | State specific objectives, including any prespecified hypotheses | 4 | In this study, we aimed to investigate factors associated with HRQoL by applying the EQ-5D-5L in a large and heterogeneous patient sample referred for treatment in specialist mental healthcare. Further, we aimed to study differences in HRQoL across mental disorders. In accordance with previous studies, we anticipated that all patient groups would score lower on HRQoL than the general population. Personality disorders were expected to report lower HRQoL than patients with anxiety disorders, depressive disorders, PTSD and trauma-related disorders, and ADHD. |
| Methods | | | |  |
| Study design | 4 | Present key elements of study design early in the paper | 4 | This study reports on data from an observational routine treatment monitoring study with a cross-sectional design. |
| Setting | 5 | Describe the setting, locations, and relevant dates, including periods of recruitment, exposure, follow-up, and data collection | 4 | Patients at a psychiatric outpatient facility in the specialist healthcare were invited to participate. (…)The period of data collection was from March 12, 2020, to November 7, 2023. |
| Participants | 6 | (*a*) *Cohort study*—Give the eligibility criteria, and the sources and methods of selection of participants. Describe methods of follow-up  *Case-control study*—Give the eligibility criteria, and the sources and methods of case ascertainment and control selection. Give the rationale for the choice of cases and controls  *Cross-sectional study*—Give the eligibility criteria, and the sources and methods of selection of participants | 4 | Cross-sectional study: Patients at a psychiatric outpatient facility in the specialist healthcare were invited to participate. (…)We included the following groups of disorders: F30 (Mood disorders), F40 (Neurotic, stress-related and somatoform disorders), F60 (Disorders of adult personality and behaviour), and F90 (Behavioural and emotional disorders with onset usually occurring in childhood and adolescence). These were labelled depressive-, anxiety, personality and hyperkinetic disorders, respectively. In addition, patients diagnosed within F43 Adjustment disorders and F44 Dissociative disorders were labelled “trauma-related disorders” |
|  |  | (*b*) *Cohort study*—For matched studies, give matching criteria and number of exposed and unexposed  *Case-control study*—For matched studies, give matching criteria and the number of controls per case |  |  |
| Variables | 7 | Clearly define all outcomes, exposures, predictors, potential confounders, and effect modifiers. Give diagnostic criteria, if applicable | 4-5 | As part of routine screening procedures, respondents self-reported if they were currently working/studying, if they recently had been or currently were on sick leave, current use of psychotropic medication, and relationship status, which was coded as being in a relationship or being single. Information regarding age, sex and psychiatric diagnoses were retrieved from clinical register data. Data on disability benefits and Work Assessment Allowance (WAA) were extracted from the Norwegian Labour and Welfare Administration (NAV). Self-report questionnaires: EQ-5D-5L, PHQ-9, GAD-7. |
| Data sources/ measurement | 8* | For each variable of interest, give sources of data and details of methods of assessment (measurement). Describe comparability of assessment methods if there is more than one group | 4-5 | Differences between groups of mental disorders on the EQ-5D index and VAS were tested with ANOVAs, with Games-Howell corrected post-hoc tests |
| Bias | 9 | Describe any efforts to address potential sources of bias | 6 | To make use of all available data, replacement of missing values was handled by multiple imputation (MI), as MI is argued to reduce bias compared to complete case analysis. (…) Initial screening of the data showed skewed values for the EQ-5D index and VAS, and the Breusch-Pagan test indicated an issue with heteroscedasticity for the regression models. We, therefore, repeated the regression analyses with robust standard errors. (…)  In addition, the regression analysis with the EQ-5D index as outcome was repeated where the PHQ-ADS variable was omitted as a covariate. |
| Study size | 10 | Explain how the study size was arrived at | 6 | To identify small effect sizes, with α = .05 and power =.80, a required sample size of N = 1200 would be needed for the ANOVAs (5 groups), and N = 878 for the regression models (12 predictors). The sample size was therefore acceptable. |

Continued on next page

| Quantitative variables | 11 | Explain how quantitative variables were handled in the analyses. If applicable, describe which groupings were chosen and why | 5-6 | See section named “Statistical analysis” |
| --- | --- | --- | --- | --- |
| Statistical methods | 12 | (*a*) Describe all statistical methods, including those used to control for confounding | 5-6 |  |
|  |  | (*b*) Describe any methods used to examine subgroups and interactions | 5-6 |  |
|  |  | (*c*) Explain how missing data were addressed | 5-6 | See Statistical analysis section and electronic supplement |
|  |  | (*d*) *Cohort study*—If applicable, explain how loss to follow-up was addressed  *Case-control study*—If applicable, explain how matching of cases and controls was addressed  *Cross-sectional study*—If applicable, describe analytical methods taking account of sampling strategy |  |  |
|  |  | (*e*) Describe any sensitivity analyses | 6 | We, therefore, repeated the regression analyses with robust standard errors (…) In addition, the regression analysis with the EQ-5D index as outcome was repeated where the PHQ-ADS variable was omitted as a covariate. |
| Results | | | | |
| Participants | 13* | (a) Report numbers of individuals at each stage of study—eg numbers potentially eligible, examined for eligibility, confirmed eligible, included in the study, completing follow-up, and analysed | 6 | . A total of 3789 responded to the invitation link, of whom 3584 consented to participate and 205 declined. Of those who consented, clinical variables from patient registers were available for 3201 patients. Patients with other disorders (n = 1190), and participants who had not completed the EQ-5D-5L (n = 64), were removed. Data from a total of 1947 (…) respondents were included in the analyses. |
|  |  | (b) Give reasons for non-participation at each stage | 6 | As above |
|  |  | (c) Consider use of a flow diagram | 6 | Due to the observational nature of the study a flow-diagram is not deemed necessary. |
| Descriptive data | 14* | (a) Give characteristics of study participants (eg demographic, clinical, social) and information on exposures and potential confounders | 6 | Information provided in text and in Table 1 and Table S1 |
|  |  | (b) Indicate number of participants with missing data for each variable of interest | Suppl | 16 (0.8 %) had one or more items missing on the EQ-5D-5L, 11 had missing on the EQ VAS, 142 (7.3%) respondents had not reported their relationship status, and five (0.3%) had missing values on PHQ-ADS. |
|  |  | (c) *Cohort study*—Summarise follow-up time (eg, average and total amount) |  |  |
| Outcome data | 15* | *Cohort study*—Report numbers of outcome events or summary measures over time |  |  |
|  |  | *Case-control study—*Report numbers in each exposure category, or summary measures of exposure |  |  |
|  |  | *Cross-sectional study—*Report numbers of outcome events or summary measures | 6-8 | Information is provided in Table 1 and Table 2 |
| Main results | 16 | (*a*) Give unadjusted estimates and, if applicable, confounder-adjusted estimates and their precision (eg, 95% confidence interval). Make clear which confounders were adjusted for and why they were included | 6-8 | Main results are reported in the Results section and in Table 3 and Table 4. |
|  |  | (*b*) Report category boundaries when continuous variables were categorized | 6-8 | As above. |
|  |  | (*c*) If relevant, consider translating estimates of relative risk into absolute risk for a meaningful time period |  | Not applicable. |

Continued on next page

| Other analyses | 17 | Report other analyses done—eg analyses of subgroups and interactions, and sensitivity analyses | 7 | See section starting with “As sensitivity analyses”. |
| --- | --- | --- | --- | --- |
| Discussion | | | | |
| Key results | 18 | Summarise key results with reference to study objectives | 9-11 | The results are summarised and discussed in the Discussion section |
| Limitations | 19 | Discuss limitations of the study, taking into account sources of potential bias or imprecision. Discuss both direction and magnitude of any potential bias | 11-12 | See section with the subheading “Limitations” |
| Interpretation | 20 | Give a cautious overall interpretation of results considering objectives, limitations, multiplicity of analyses, results from similar studies, and other relevant evidence | 9-11 | The interpretation of the findings is provided in the Discussion section. |
| Generalisability | 21 | Discuss the generalisability (external validity) of the study results | 9-11 | The generalisability of the study results are addressed in the Discussion section. |
| Other information | |  | | |
| Funding | 22 | Give the source of funding and the role of the funders for the present study and, if applicable, for the original study on which the present article is based | 11 | See information provided under the subheading “Funding”. |

*Give information separately for cases and controls in case-control studies and, if applicable, for exposed and unexposed groups in cohort and cross-sectional studies.

**Note:** An Explanation and Elaboration article discusses each checklist item and gives methodological background and published examples of transparent reporting. The STROBE checklist is best used in conjunction with this article (freely available on the Web sites of PLoS Medicine at http://www.plosmedicine.org/, Annals of Internal Medicine at http://www.annals.org/, and Epidemiology at http://www.epidem.com/). Information on the STROBE Initiative is available at www.strobe-statement.org.
