# Supplementary material for: Health-related quality of life in psychiatric outpatients: a cross-sectional study of associations with symptoms, diagnoses, and employment status
Source: Qual Life Res. 2024 Aug 7;33(11):3093–105. doi: 10.1007/s11136-024-03748-3 (PMC11541330; doi:10.1007/s11136-024-03748-3)
Supplement: Supplementary file 2 — Supplementary Material 2 [file 11136_2024_3748_MOESM2_ESM.docx]

**Supplementary materials.**

**Table S1.**

*EQ-5D index and EQ VAS scores for separate primary diagnoses.*

| **Diagnosis** | ***n*** | **EQ-5D index** | **EQ VAS** |
| --- | --- | --- | --- |
|  |  | M (SD) | M (SD) |
| F30 | 132 | 0.50 (0.25) | 51.48 (19.31) |
| F32 | 619 | 0.44 (0.23) | 45.17 (17.79) |
| F400 | 35 | 0.40 (0.23) | 43.86 (18.22) |
| F401 | 103 | 0.51 (0.23) | 49.42 (17.72) |
| F410 | 32 | 0.52 (0.23) | 56.16 (19.95) |
| F411 | 185 | 0.48 (0.22) | 47.45 (17.96) |
| F412 | 46 | 0.56 (0.21) | 52.20 (16.14) |
| F413 | 10 | 0.34 (0.34) | 49.60 (16.70) |
| F419 | 18 | 0.48 (0.20) | 51.61 (20.18) |
| F42 | 13 | 0.51 (0.29) | 54.62 (21.12) |
| F43 | 216 | 0.40 (0.26) | 44.40 (18.98) |
| F45 | 29 | 0.35 (0.30) | 45.82 (20.88) |
| F603 | 57 | 0.36 (0.26) | 42.53 (19.38) |
| F606 | 58 | 0.42 (0.26) | 44.22 (17.17) |
| F61 | 29 | 0.38 (0.26) | 40.79 (18.40) |
| F90 | 320 | 0.53 (0.25) | 51.55 (19.90) |

*Note*. Only diagnostic subgroups with at least *n* = 10 are shown.

**Analysis of missing data patterns**

We systematically explored missing data patterns by summarizing variables with missing data. Table S2 shows the distribution of missing data.

**Table S2.**

*Distribution of Missing Data*

| Variable | Missing data |
| --- | --- |
|  | n (%) |
| EQ-5D index | 16 (0.8) |
| EQ-5D VAS | 11 (0.6) |
| Relationship status | 142 (7.3) |
| PHQ-ADS | 5 (0.3) |

Little’s Missing Completely at Random (MCAR) test was used to identify missing data patterns, with the null hypothesis that data is missing completely at random. The analysis did not reject the null hypothesis; *χ2* = 78.9, *df* = 74, *p* = 0.326 and the data was assumed to be missing at random. To make use of all data points, we chose to replace missing values by multiple imputation, as this approach is preferred over listwise deletion, also when data are MCAR (55-57). The MICE (Multivariate Imputation by Chained Equations) (58) package for the statistical environment of *R*, with 200 imputed datasets and 10 iterations was applied. As recommended by Sterne (56), we included a wide range of variables in the imputation model: EQ-5D-5L index, EQ-5D VAS, age, sex, relationship status, comorbidity, occupation status, diagnostic grouping, use of medication, and PHQ-ADS.
